# Effect of DS Concentration on the PRO Performance Using a 5-Inch Scale Cellulose Triacetate-Based Hollow Fiber Membrane Module

**DOI:** 10.3390/membranes8020022

**Published:** 2018-05-01

**Authors:** Masahiro Yasukawa, Daisuke Shigefuji, Masafumi Shibuya, Yuki Ikebe, Ryuto Horie, Mitsuru Higa

**Affiliations:** 1 Graduate School of Sciences and Technology for Innovation, Yamaguchi University, Yamaguchi 755-0097, Japan; myasu@yamaguchi-u.ac.jp; 2 Graduate School of Science and Engineering, Yamaguchi University, Yamaguchi 755-0097, Japan; r016vh@yamaguchi-u.ac.jp (D.S.); glory.color413@gmail.com (M.S.); u003vh@yamaguchi-u.ac.jp (Y.I.); s022fj@yamaguchi-u.ac.jp (R.H.)

**Keywords:** pressure-retarded osmosis, hollow fiber membrane, pilot-scale

## Abstract

In this study, pressure-retarded osmosis (PRO) performance of a 5-inch scale cellulose triacetate (CTA)-based hollow fiber (HF) membrane module was evaluated under a wide range of operating conditions (0.0–6.0 MPa of applied pressure, 0.5–2.0 L/min feed solution (FS) inlet flow rate, 1.0–6.0 L/min DS inlet flow rate and 0.1–0.9 M draw solution (DS) concentration) by using a PRO/reverse osmosis (RO) hybrid system. The subsequent RO system for DS regeneration enabled the evaluation of the steady-stated module performance. In the case of pilot-scale module operation, since the DS dilution and the feed solution (FS) up-concentration had occurred and was not negligible, unlike the lab-scale experiment, PRO performance strongly depended on operating conditions such as inlet flow rates of both the DS and FS concentration. To compare the module performance with different configurations, we proposed a converted parameter in which a difference of the packing density between the spiral wound (SW) and the HF module was fairly considered. In the case of HF configuration, because of high packing density, volumetric-based performance was higher than that of SW module, that is, the required number of the module would be less than that of SW module in a full-scale PRO plant.

## 1. Introduction

Salinity gradient power (SGP) is a renewable energy resource [1,2,3,4,5]. It is available when salt water and fresh water mix to form a brackish solution. One of the emerging technologies that enables the extraction of SGP is pressure-retarded osmosis (PRO) power generation [6,7,8,9,10], which converts the energy of the salinity gradient between a draw solution (DS, e.g., seawater) and a feed solution (FS, e.g., fresh water) to electricity by using a semipermeable membrane. The PRO technology, pioneered by Loeb [7,8], has received significant interest in the past. In late 2009, Statkraft, a Norwegian energy company, built the world’s first PRO osmotic power plant [11]. According to Statkraft’s projection, the PRO technology will be profitable provided its power density can reach 5 W/m^2^ or above [12,13]. Although the Statkraft Company has already stopped their PRO projection, progress for its commercialization has been continued by other researchers and companies.

Up to now, many studies have investigated the PRO-performance using flat-sheet membrane coupons [14,15,16,17,18,19,20,21,22,23] and hollow-fiber (HF) membranes [24,25,26]. In 1970s, Loeb et al. [24] reported on PRO experiments using an aromatic polyamide-based HF seawater reverse osmosis (SWRO) membrane (Du Pont Permasep B-10) enclosed in a “minipermeator,” which contained 150 fibers with the length <76 cm. They used diluted Dead Sea brine and distilled water as permeate-receiver and permeate-donor, respectively, and then proposed that 0.07 dollars per kilowatt hour in a PRO power plant could be produced. In recent years, because Hydration Technology Innovation (HTI, Albany, OR, USA) developed and commercialized flat-sheet cellulose triacetate (CTA) FO membranes and a thin-film composite (TFC) FO membrane, many researchers have accessed these commercial FO membrane coupons and used them for laboratory-scale PRO studies whose effective membrane areas were 18.75 [16], 20.02 [18], and 140 cm^2^ [17]. Achilli et al. [16] reported that 5.06 W/m^2^ of power density was obtained when the osmotic pressure difference was 4882 kPa under a hydraulic pressure difference of 972 kPa. In addition to the commercial membrane, some researchers also have developed PRO membranes by themselves. N.Y. Yip et al. [14] fabricated a flat-sheet thin-film composite (TFC) membrane and then evaluated the PRO performance with 20.02 cm^2^ of the active area. Although their fabricated PRO membrane performance was not examined under a high pressurized condition, their membrane achieved the power density of about 10 W/m^2^ when using seawater and river water as DS and FS, respectively, on their calculation. Chou et al. [25] also prepared TFC HF membranes and then estimated the PRO performance using a self-made HF mini-module that consisted of 15 HF membranes with an effective length of 28 cm. Their PRO membrane achieved a power density of 10.6 W/m^2^ when using 1.0 M NaCl aq and DI water as DS and FS, respectively. The recent trend of using PRO performance in developing membranes is summarized in the literature [27]. These investigations on PRO performance using small flat-sheet membrane coupons or miniature HF membrane modules in lab-scale are important to demonstrate the PRO analysis and to develop the PRO membrane.

On the other hand, to design the full-scale PRO plant, module-scale analyses have been also recommended because the module performance strongly depends on the operating condition and module configuration as well as the membrane performance. However, the module-scale analysis has been rarely compared with the lab-scale analysis because the number of available PRO modules is still limited. Xu et al. [28] performed a PRO performance test to evaluate the effect of operating conditions on the permeate flux in the FO and PRO processes using a spiral wound (SW) FO membrane module of Hydrowell^®^ (HTI), which has a total effective membrane area of 0.94 m^2^. Their results indicated that the module performance dramatically decreased as compared with those of the lab-scale flat FO coupon, due to DS dilution effect and mass transfer resistance. Kim et al. [29] also evaluated the prototype SW PRO membrane module performance with an effective membrane area of 29 m^2^. The power density of the module was about 1.0 W/m^2^ at the hydraulic pressure difference of 9.8 bar when using 0.6 M NaCl and tap water as DS and FS, respectively.

In addition to SW module configuration, HF module configuration has also been investigated under PRO operation. However, the HF module analysis is quite rare when compared with the SW module analysis. Saito et al. demonstrated the PRO test using brine from a seawater RO plant and treated water from a regional sewage treatment facility as DS and FS, respectively. However, their evaluation of PRO performance was carried out under limited conditions: a constant osmotic pressure difference between DS and FS and a small range of hydraulic pressure differences (25–29 bar). Therefore, to understand the role of the various operational parameters on the module flux performance in the PRO mode, the PRO performance of a prototype module at various operating conditions needs to be evaluated. To the author’s best knowledge, a systematic evaluation of prototype-scale PRO membrane modules with large area is still not available.

The purpose of this study was to evaluate the PRO performance of a pilot-scale PRO HF module with an effective area exceeding 70 m^2^ under wide range operational conditions (e.g., inlet flow rates of DS and FS, applied pressure differences and DS concentrations). To evaluate the steady state HF module performance, a PRO/RO hybrid system in which the RO system as DS regeneration system [30] was used in this study. We investigated the effect of operational parameters such as DS and FS inlet flow rates, DS concentration, and applied hydraulic pressure on the module performance. Furthermore, the PRO performance of the CTA-HF module was then compared with those of the SW modules reported in literature that considered membrane area-based and volumetric-based power density [31].

## 2. Experimental

### 2.1. Chemicals

For the module performance test, sodium chloride (NaCl, Nacalai Tesque Co., Ltd., Kyoto, Japan) was used to prepare the salt solution used as the DS. Tap water was filtered by an activated carbon filter to remove chlorine before use, and then used as the FS and DS solvent. The conductivity of the tap water was about 0.28 mS/cm.

### 2.2. HF Membrane Module

A CTA-HF membrane module used in this study was designed for FO and PRO applications and kindly provided from TOYOBO Co. Ltd., Japan. Table 1 shows the specifications of the test module. A large number of fine HFs (inner diameter: 60 μm, and outer diameter: 160 μm) were bundled as the element (diameter: 136 mm, length: 683 mm) and installed inside the module (diameter: 176 mm, length: 825 mm). This prototype CTA-HF module has a large membrane area of approximately 72 m^2^. The module already has four open ports, corresponding to the inlets and outlets for DS and FS, respectively. The HF has an outer active layer surface [32] and therefore DS and FS were introduced to the shell-side and the lumen-side of the HF membranes, respectively. The DS flow was streamed from the central core tube, where the HF bundles were crossly wounded, into the radial flow direction as shown in our previous literature [30].

### 2.3. CTA HF Module Performance

#### 2.3.1. PRO/RO Hybrid System

**Figure 1:**
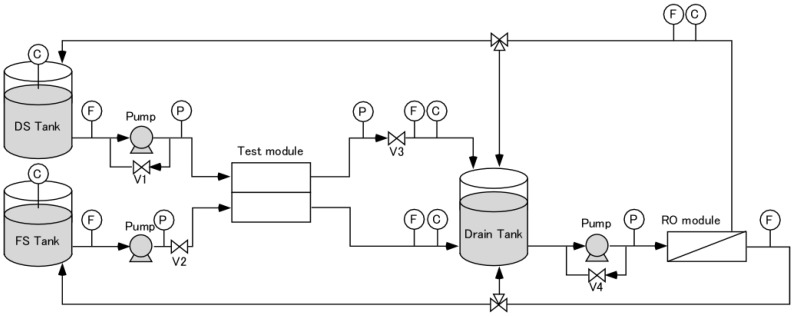
A schematic diagram of the bench-scale pressure-retarded osmosis (PRO) evaluation system. The character inside the circles denote the following: C, conductivity meter; F, flow meter; P, pressure gauge.

Figure 1 shows a schematic diagram of the PRO/RO hybrid system for the HF module performance analysis [31]. This system consisted of a PRO evaluation unit and a DS-regeneration unit by RO. To perform a PRO test at the steady state during the measurement, the diluted DS and concentrated FS were poured into the drain tank and then regenerated to the initial concentration of DS and FS using the DS-regeneration unit by RO. The DS-regeneration unit consists of an RO module (SWC1-4040, Hydronautics, Oceanside, CA, USA) and a high-pressure pump (N-28CB-206K1, Capital Industry Co., Ltd., Tokyo, Japan). The PRO evaluation unit consists of DS and FS tanks, a test module, and liquid-supplying pumps. A high-pressure pump (N-28CB-206K1, Capital Industry Co., Ltd., Japan) and a low-pressure pump (TYP-2800, Deng Yuan industrial Co., Ltd., Taichung City, Taiwan) were used to supply FS and DS, respectively. The DS inlet pressure, DS outlet pressure and FS inlet pressure (*P*_FS,in_) were measured using a pressure gauge (KH15-883, Nagano Keiki Co., Ltd., Tokyo, Japan). The DS inlet flow rate (*Q*_DS,in_), DS outlet flow rate (*Q*_DS,out_), FS inlet flow rate (*Q*_FS,in_) and FS outlet flow rate (*Q*_FS,out_) were measured by using flow meters (FDM-5AY, Keyence corporation, Osaka, Japan). The DS inlet concentration (*C*_DS,in_), DS outlet concentration (*C*_DS,out_), FS inlet concentration and FS outlet concentration (*C*_FS,out_) were also measured using ionic conductivity meters (EC430, Suntex Instruments Co. Ltd., New Taipei City, Taiwan). These all data were monitored on the real time by using multi data logging system (GL820-UM-801, Graphtec Co., Ltd., Yokohama, Japan) connected with a personal computer in the time frequency of 1/10 s^−1^.

#### 2.3.2. RO Experiments

To estimate the water permeability and salt permeability of the test module, a RO mode test was preliminarily carried out by closing valve V2 of the PRO evaluation system, that is, the permeated water flowed out from the outlet of the lumen FS-side. The permeate flow rate (*Q*_w_) and RO flux (*J*_w_) was calculated using effective membrane area (*S*_m_) and obtained data (*Q*_FS,in_ and *Q*_FS,out_) as follows:

(1) $$Q_{w}\;=\;Q_{\mathrm{FS},\mathrm{in}}\;-\;Q_{\mathrm{FS},\mathrm{out}}$$

(2) $$J_{w}^{\mathrm{RO}}\;=\;60Q_{w}/S_{m}$$

The water permeability (*A*) was then calculated as follows:

(3) $$J_{w}^{\mathrm{RO}}\;=\;A\left(\Delta \pi_{\mathrm{ave}}\;-\;\Delta P_{\mathrm{ave}}\right)$$

where Δ*π*_ave_ and Δ*P*_ave_ are averages of the osmotic and applied hydraulic pressure differences between the inlet and outlet, respectively, between the DS and FS sides. The flow rate of FS on the high-pressure side was maintained constant at 4.0 L/min during the RO test. Water permeability (*A*) was estimated from the slope of the *J*_w_—Δ*P* curve plotted using Equation (3) when deionized water was used as the feed solution of the high-pressure side (Δ*π* = 0). Salt rejection (*R*) was also calculated using 8.6 mM, 100 mM, and 200 mM NaCl solution as the feed as follows [16]:

(4) $$R=\;\frac{C_{P}}{C_{F,ave}}$$

where *C*_F_,_ave_ is the average of the salt concentration of the feed solution between the inlet and outlet. *C*_P_ is the salt concentration of the permeate. The salt permeability (*B*) of the test module was then calculated as follows [16,33]:

(5) $$B=\frac{A\left(1-R\right)\left(\Delta P_{ave}-\Delta \pi_{ave}\right)}{R}$$

where Δ*P*_ave_ and Δ*π*_ave_ are averages of the hydraulic and osmotic pressure differences, respectively, between the inlet and outlet.

#### 2.3.3. PRO Performance Evaluation

To evaluate the PRO performance, the PRO/RO hybrid system was operated under various operating conditions. Here, we investigated the PRO module performance under a wide range of operating conditions: *C*_DS,in_ of 0.1 ~ 0.9 M, the hydraulic pressure in 0 ~ 60 bar, *Q*_FS,in_ in 0.5 ~ 2.0 L/min and *Q*_DS,in_ in 1.0 ~ 6.0 L/min. The water flux and power density of the test module were then calculated from the monitored data. The temperature was maintained at 26 ± 1 °C during all experiments.

In the case of a pilot-scale module experiment, the FS up-concentration and DS dilution must have occurred and are not negligible unlike the lab-scale experiment. Therefore, to estimate the effect of FS up-concentration, the permeation rate (*η*) was defined as follows [31]:

(6) $$\eta \equiv \frac{Q_{FS,in}-Q_{FS,out}}{Q_{FS,in}}\times 100$$

When *η* = 100, the entire FS-side solution permeates through the membrane to the DS side. To estimate the effect of DS dilution, the dilution factor (*Φ*) is also defined for as follows [31]:

(7) $$\Phi \equiv \frac{Q_{w}-Q_{DS,in}}{Q_{DS,in}}$$

When *Q*_DS,in_ is much larger than the permeate flow rate, the *Φ* approaches 1, which means that *C*_DS,out_ is approximately equal to *C*_DS,in_. In addition, the PRO module should be operated at the *Φ* less than 2 because the effective osmotic pressure would be disappeared within the module under the optimum pressure difference condition (Δ*P* = *π*/2), that is, the *C*_DS,out_ would become less than half of *C*_DS,in_, resulting in the substantial flux reduction [28].

To compare the PRO membrane performance in lab-scale, membrane area-based power density (*W*^area^), defined as the calculated power output per unit membrane area, is often used as follows [31,34]:

(8) $$W^{\mathrm{area}}\;=\;J_{w}\Delta P\;=\;A\left(\Delta \pi \;-\;\Delta P\right)\Delta P$$

On the other hand, in the case of module performance comparison, membrane area-based power density (*W*^area^) would not be suitable as a comparable parameter for fair PRO performance comparison [31]. In the case of current RO module case, the water permeability of CTA-based HF membrane was about 1/10th that of polyamide (PA)-based TFC membrane. However, CTA-HF module has also been succeeded its commercialization same to PA-based SW module because an HF membrane module has approximately 10 times higher packing density (membrane area per unit volume) than that of a SW membrane module [31]. Hence, the water productivities (water flux per unit volume of the module) of CTA HF RO module and SW TFC RO are consequently comparable. From the industrial point of view, it is important for a commercially feasible PRO plant to compare the power density not by power per membrane area but by power per unit volume of a membrane module, because the volume of the module as well as the performance and cost per module are also important factors in the power cost of the PRO plant. Therefore, the permeate water flux per unit volume of a module (*J*_w_^vol^) was obtained as follows [31]:

(9) $$W^{\mathrm{area}}\;=\;J_{w}\Delta P\;=\;A\left(\Delta \pi \;-\;\Delta P\right)\Delta P$$

The power density per unit volume of a module (*W*^vol^) is then calculated as follows [31]:

(10) $$W^{vol}\;=\;Q_{w}/V_{\mathrm{mod}}$$

where *V*_mod_ is the volume of the module.

#### 2.3.4. Module Performance Comparison between SW and HF Configuration

In PRO experiments in literature, almost all researchers compared their data with power density, which is defined as the calculated power per unit membrane area of flat-sheet membrane coupons [14,15,16,17,18,19,20,21,22,23]. There are few studies on PRO performance using a membrane module, especially using a HF membrane module. To compare the PRO performance of the HF PRO module in the current study with that of the flat-sheet membrane coupons in literature, we also propose a converted coefficient. As consequence, the conversion coefficient *γ*_SW_^HF^ is given as follows:

(11) $$\gamma_{SW}^{HF}=\;\frac{1}{\alpha_{SW}}\frac{\beta_{HF}}{\beta_{SW}}$$

where *α*_SW_ (=*β*_SW_/*β*_SW(PRO)_) is the module factor in a spiral wound (SW) configuration, which is the packing density ratio between the standard SW module and the current developing SW module for PRO. *β*_SW_ and *β*_HF_ are the packing density (*S*_m_/*V*_mod_) of the SW and HF modules, respectively. For simplicity, here, the *γ*_SW_^HF^ was calculated under an assumption that *α*_SW_ was equal to 1. The volume (*V*_mod_) and area (*S*_m_) of a typical 8-in SW module were set to 0.033 m^3^ and 40 m^2^, respectively, and used as a standard SW module for comparison. Therefore, the packing density (*S*_m_/*V*_mod_) of the standard SW module (1215 m^2^/m^3^) was estimated higher than the current developing SW module (920–990 m^2^/m^3^ [28,29]) for fair comparison. On the other hand, those of the 5-in HF module used in the current study were 0.0093 m^3^ and 72 m^2^, respectively. Based on these values, the HF module may be assumed that if it has a SW configuration, the membrane area of the module can be assumed to be approximately 11 m^2^. Consequently, the value of *γ*_SW_^HF^ was set as:

(12) $$\gamma_{SW}^{HF}\approx \;\frac{\beta_{HF}}{\beta_{SW}}=\frac{S_{m\left(HF\right)}}{V_{mod\left(HF\right)}}\frac{V_{mod\left(SW\right)}}{S_{m\left(SW\right)}}=\frac{72}{0.0093}\frac{0.033}{40}=6.4$$

The converted water flux (*J*_w_^conv^) is then calculated using the following equation:

(13) $$J_{w}^{conv}=\;\gamma_{SW}^{HF}J_{w}=A^{conv}\left(\Delta \pi -\Delta P\right)\Delta P$$

where *A*^conv^ is defined as

(14) $$A^{conv}\equiv \gamma_{SW}^{HF}A$$

The converted *B* value (*B*^conv^) is expressed in terms of the following equation:

(15) $$B^{conv}=\gamma_{SW}^{HF}B$$

The converted power density (*W*^conv^) is calculated using the following equation:

(16) $$W^{conv}=\;\gamma_{SW}^{HF}W^{area}=A^{conv}\left(\Delta \pi -\Delta P\right)\Delta P$$

Equation (16) indicates that *W*^conv^ has the maximum value (*W*_conv_^max^) at Δ*P* = Δ*π*/2:

(17) $$W_{max}^{conv}=A^{conv}{\left(\Delta \pi /2\right)}^{2}$$

## 3. Results and Discussion

### 3.1. Water and Salt Permeability in the RO Experiment

**Figure 2:**
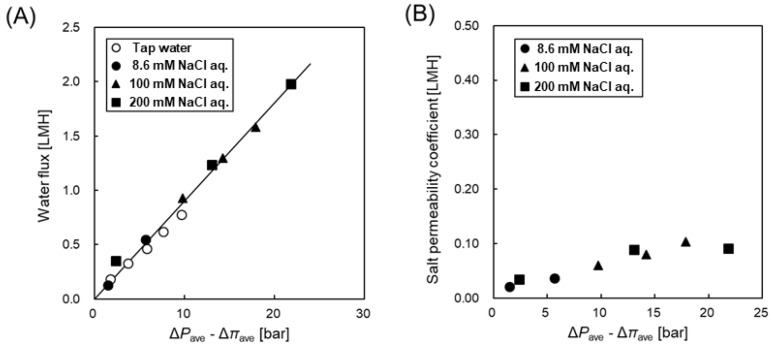
(**A**) Water flux and (**B**) salt permeability coefficient as a function of Δ*P*_ave_-Δ*π*_ave_ with different salt concentration condition.

Figure 2A shows water flux (*J*_w_) as a function of the difference between average hydraulic and osmotic pressure (Δ*P*_ave_-Δ*π*_ave_) in the RO mode with different salt concentrations (8.6 mM, 100 mM, 200 mM NaCl aq. and tap water). Although the salt concentration conditions were different, the *J*_w_ increased with increasing driving force, and agreed with a single linear line by adopting the average values of Δ*P* and Δ*π* within the module. The maximum flux in the RO experiment was about 2 LMH (2.4 L/min) and this value was about 60% of the feeding flow rate (4 L/min). The concentration difference between the inlet and outlet was considered by using the average value of them in Equations (4) and (5). The water permeability of the test HF module was calculated using by the slope of this line with Equation (3) and was shown to be 0.08 LMH/bar. Therefore, the converted water permeability (*A*^conv^) of this module can be assumed to 0.51 LMH/bar by considering the difference between the HF and SW module configurations. On the other hand, the water permeability of the commercial flat-sheet CTA-FO membrane (HTI) ranges from 0.28 to 0.50 LMH/bar as shown in the literature [35]. Hence, the converted water permeability of the CTA-HF test module is almost of the same to that of the commercial flat-sheet CTA membrane, that is, the productivity of this CTA HF module is almost the same when compared to the SW module with the commercial flat-sheet CTA membrane.

In the case of the salt permeability coefficient (*B*) as shown in Figure 2B, although the obtained *B* value slightly depended on the Δ*P*_ave_-Δ*π*_ave_, the *B* value was from 0.02 to 0.10 LMH. Therefore, the converted *B* value (*B*^conv^) of the test module was from 0.13 to 0.65 LMH. Because the *B* values of the commercial flat sheet CTA membrane are in the range from 0.15 LMH to 0.44 LMH as shown in a literature [35], the *B*^conv^ of the test HF module was a little higher than that of the commercial flat-sheet CTA membrane when considering the difference between the HF and SW module configuration.

### 3.2. PRO Performance

#### 3.2.1. Effects of *Q*_FS,in_ and *Q*_DS,in_ on Water Flux

**Figure 3:**
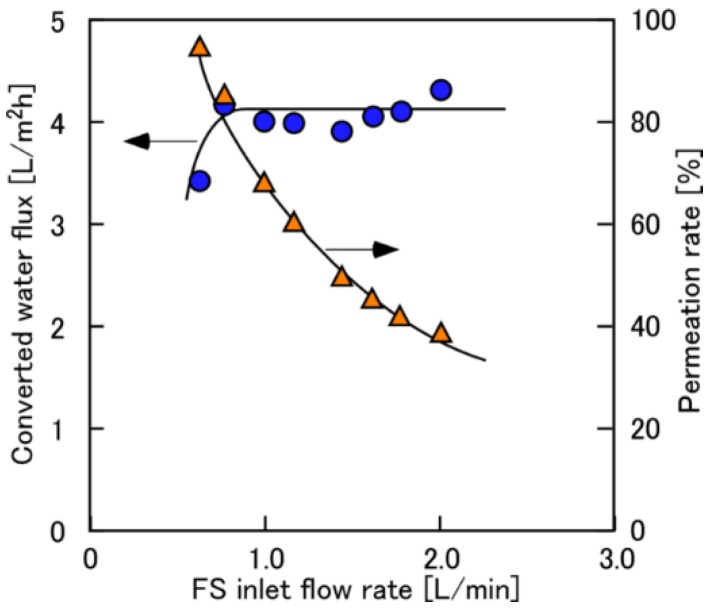
Converted water flux and permeation rate as a function of *Q*_FS,in_. PRO conditions: Δ*P*, 11 bar; DS, 0.6 M NaCl; FS, tap water; *Q*_DS,in_: 4.0 L/min.

Figure 3 shows the converted permeate water flux and the *η* as a function of *Q*_FS,in_ when using 0.6 M NaCl as DS at Δ*P* = 11 bar. The *η* reached 100% when the *Q*_FS,in_ was less than 0.6 L/min, which means all feeding FS was penetrated through the membrane module from DS to FS. When the *Q*_FS,in_ > 0.8 L/min (*η* < 80%), the water flux had a constant value of approximately 4.0 L/(m^2^h) of the converted *J*_w_. Therefore, this constant value suggested that the effect of ECP on the membrane surface of the FS side (the lumen side) can be negligible when the *Q*_FS,in_ > 0.8 L/min. In other words, ECP can be negligible even at the high *η* (=80%), which would be the preferable operating condition due to low FS pumping energy.

**Figure 4:**
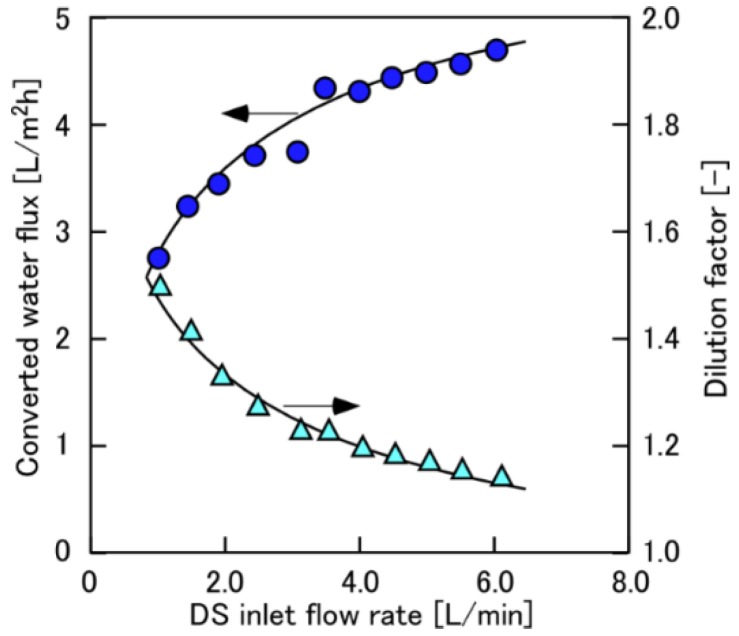
Converted water flux and dilution factor as a function of draw solution (DS) flow rate. PRO conditions: Δ*P*, 11 bar; DS, 0.6 M NaCl; FS, tap water; *Q*_FS,in_: 1.5 L/min.

Figure 4 shows the converted water flux and the *Φ* as a function of *Q*_DS,in_ when using 0.6 M NaCl as DS at Δ*P* = 11 bar. The *Φ* decreased from 1.5 to 1.1 with increasing *Q*_DS,in_ from 1.0 to 6.0 L/min. Since the water flux also increased with decreasing *Φ*, the effects of ECP (and also DS dilution) were not completely suppressed even at the high *Φ* of 1.1 (*Q*_DS,in_ = 6 L/min which was the maximum flow rate of this evaluation system because of pump limitation). The similar trends in which the *η* less than 80% was sufficient to obtain the constant water flux whose data are not shown here were also obtained when changing the DS concentrations. Hereafter, the constant values at the high feeding flow rates of DS and FS (the *Q*_DS,in_ and *Q*_FS,in_ were 4 and 1.5 L/min, respectively) were used to discuss the DS concentration effect.

#### 3.2.2. Effects of Applied Hydraulic Pressure and DS Concentration

**Figure 5:**
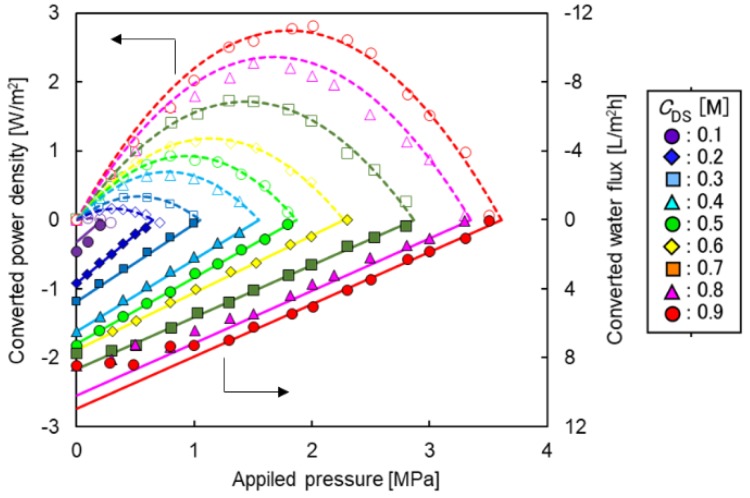
Converted power density and water flux as a function of applied hydraulic pressure with different DS concentrations (*C*_DS_). PRO conditions: FS, tap water; *Q*_DS,in_: 4.0 L/min; *Q*_FS,in_; 1.5 L/min.

Figure 5 shows the converted water flux and the converted power density as a function of the Δ*P* with different *C*_DS,in_. To discuss both water flux and power density simultaneously, the *y*-axis for the water flux had an opposite direction (upper was minus direction). As shown in Equation (3), the water flux decreased with increasing Δ*P* and increased with increasing *C*_DS,in_. The optimum Δ*P* for the maximum power density was also increased with increasing *C*_DS,in_. When the Δ*P* was relatively high compared to the osmotic pressure difference, the water flux decreased linearly with increasing Δ*P* according to Equation (3). On the other hand, when the Δ*P* was relatively low compared to the osmotic pressure difference and the *C*_DS,in_ was high, the water flux did not agreed with this linear trend because of high *η* and/or high *Φ* (the water flux strongly influenced the DS dilution and FS up-concentration). Therefore, linear approximation was adjusted by using the data with the relative low water flux at the relative high Δ*P*. In this operating condition, the *η* and *Φ* were sufficiently low, that is, the water flux was not influenced by the inlet flow rates. The Δ*P* at which the water flux and power density intersected can be assumed to be the effective osmotic pressure difference, Δ*π*_eff_, under the PRO operation (Δ*P* = Δ*π*_eff_).

**Figure 6:**
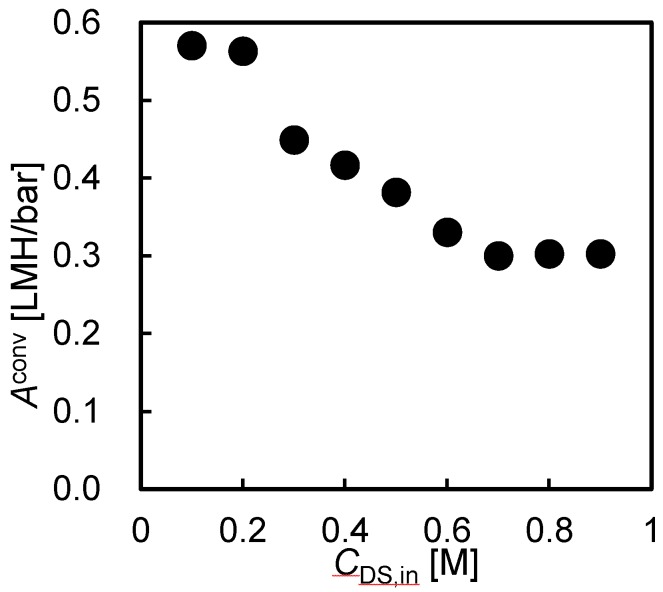
Converted water permeability as a function of applied hydraulic pressure at various DS concentrations (*C*_DS_). PRO conditions: FS, tap water; *Q*_DS,in_: 4.0 L/min; *Q*_FS,in_; 1.5 L/min.

Figure 6 shows the converted water permeability, *A*^conv^, as a function of Δ*P* with different *C*_DS,in_. Although Equation (3) does not indicate the *C*_DS,in_ dependence on the *A*^conv^, the *A*^conv^ clearly decreased with increasing *C*_DS,in_. Because the water flux was almost 0 when Δ*P* = Δ*π*_eff_, this trend was mainly due to an internal concentration polarization (ICP) involving the DS leakage from DS to FS. When both *C*_DS,in_ and Δ*P* were high, the *A*^conv^ became half compared to the those with lower *C*_DS,in_ and Δ*P*. Whereas, when the both *C*_DS,in_ and Δ*P* were low, the *A*^conv^ was almost the same as the original *A*^conv^ obtained by the RO experiment. This also supported the hypothesis that the unfavorable reduction in *A*^conv^ was due to the DS leakage and subsequent ICP.

#### 3.2.3. Deviations from the Theory

**Figure 7:**
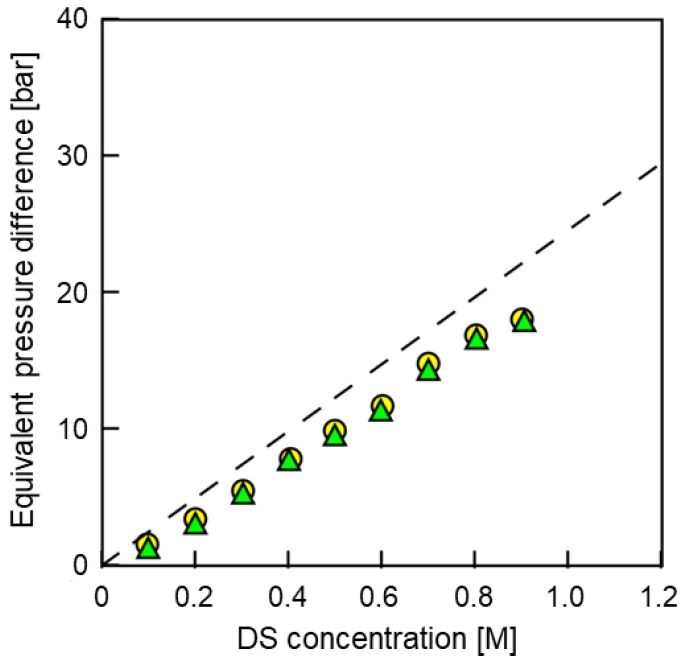
Equivalent pressure differences in the PRO process as a function of CDS. Circles, the applied pressure difference at the maximum power density; triangles, the half value of Δ*P* at zero water flux obtained from the *J*_w_-Δ*P* curve (1/(2Δ*P*_Jw=0_)); dashed line, the half value of Δ*π* between DS and FS (Δ*π*/2). PRO conditions: FS, tap water; *Q*_DS,in_: 4.0 L/min; *Q*_FS,in_; 1.5 L/min.

Figure 7 shows the Δ*π*_eff_ as a function of *C*_DS_. In this figure, Δ*P*_Wmax_ (〇) indicates the applied pressure difference at the maximum power density; 1/(2Δ*P*_Jw=0_) (▲) indicates the half value of Δ*P* when the *J*_w_ became zero obtained from Figure 6; Δ*π*/2 (dashed line) indicates the half value of the theoretical Δ*π* between DS and FS calculated from van’t Hoff equation [36]. Results indicated that the Δ*P*_Wmax_ and 1/(2Δ*P*_Jw=0_) were almost the same values as each other and approximately 70–85% of theoretical Δ*π*/2. Since the *η* and *Φ* was sufficiently low due to the low water flux, the deviations of the slope in Figure 7 between the Δ*π*/2 and the others were mainly due to the ICP. The same value of Δ*P*_Wmax_ and 1/(2Δ*P*_Jw=0_) indicated that the pressure dependence of the salt permeability was quite low, as shown in Figure 2.

**Figure 8:**
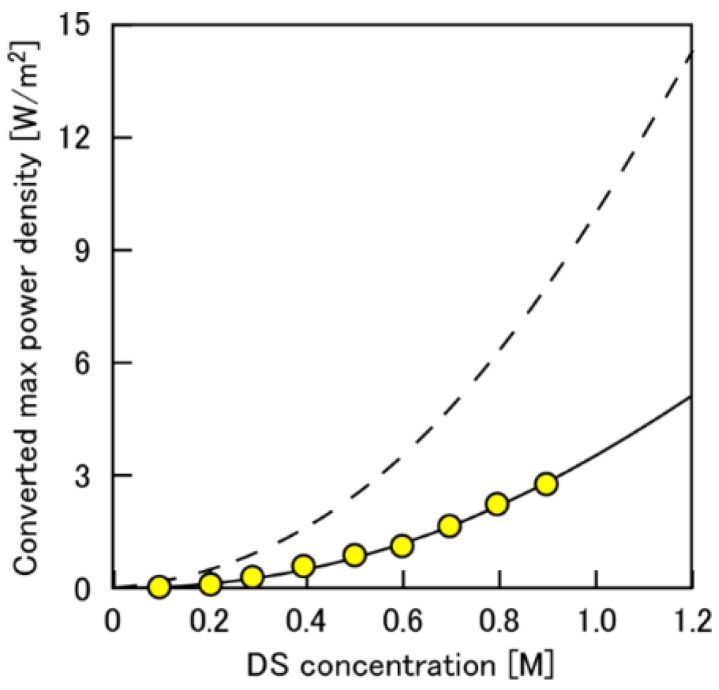
Converted maximum power density as a function of DS concentration. The broken curve represents the theoretical values; circles represent the experimental values. PRO conditions: FS, tap water; *Q*_DS,in_: 4.0 L/min; *Q*_FS,in_; 1.5 L/min.

Figure 8 shows the converted maximum power density as a function of *C*_DS_. Equation (15) indicates that the power density is on the curve of (*C*_DS_)^2^:

(18) $$W_{max}^{conv}=A^{conv}{\left(\Delta \pi /2\right)}^{2}=\;A^{conv}{\left(\mathrm{RT}\right)}^{2}{\left(C_{DS}\right)}^{2}$$

In the figure, the circle plots show the experiment data, and the dashed curve shows the simulated converted power density calculated using *A*^conv^ converted from the water permeability obtained by the RO test. The solid curve is obtained by fitting Equation (18) with the experiments, using *A*^conv^ as a fitting parameter. Interestingly, the experimental result agreed well with the simple approximation using the apparent converted water permeability (*A*^conv^_app_) at a wide range of DS concentrations despite the lack of consideration about the other complex effects such as DS dilution, ICP and so on. Therefore, this *A*^conv^_app_ can be used as a useful comprehensive parameter, which includes the total effects, such as ICP within the membrane, DS dilution within the module, pressure drop distribution and so on, for estimating the module performance with different DS concentrations. Xu et al. [28] also showed a similar trend, that there was a relative linear relationship between *C*_DS_ and osmotic-driven water flux of the module, and also showed that the water flux of the module was lower than those of the small size membrane coupon due to the DS dilution and ICP. In our case, the *A*^conv^_app_ obtained by the fitting was 0.18 LMH/bar and approximately 1/3rd of the water permeability obtained by the RO mode, 0.51 LMH/bar. Therefore, since the dilution effect on the PRO performance was almost negligible as shown in Figure 5, the difference between *A*^conv^_app_ in RO and *A*^conv^_app_ in PRO was mainly due to the ICP effect, although the ECP effect still remained to some extent.

### 3.3. Module Performance Comparison in PRO with Different Configurations

Table 2 shows a comparison between the PRO module performance with different configuration such as SW and HF. To the authors’ best knowledge, there are few reports on the comparison of the module-based PRO performance because it is difficult to fairly compare their performance when considering different module configurations. Xu [28] et al. evaluated the PRO performance using an SW-FO membrane module (Hydrowell^®^ from HTI Company, Albany, OR, USA) based on a CTA membrane with the water permeability of 0.79 LMH/bar. They reported that the *W*_max_^area^ of 0.4 W/m^2^ was obtained at the pressure difference of 4.5 bar when using 0.5 M NaCl as DS. Kim et al. [29] also evaluated the PRO performance using a prototype SW PRO membrane module based on a TFC membrane with the water permeability of 0.66–0.81 LMH/bar. The power density of the module was about 0.81 W/m^2^ at the hydraulic pressure difference of 9.8 bar when using 0.52 M NaCl and tap water as DS and FS, respectively. In these cases, the membrane area-based PRO module performances were directly able to be compared because these module configurations were the same. The PRO performance of the latter module was higher than those of the former due to the highly applied hydraulic pressure.

However, to compare the PRO performances with different configuration such as SW and HF, a volumetric-based comparison is needed as well as a membrane area-based comparison. Therefore, we also estimated the volumetric-based power density as shown in Table 2 using the module dimension information in the literature [29] for the latter SW PRO module. In the case of the former SW FO module, unfortunately, there was no description on the volume of the module. Hence, here, in order to compare their performance with the other modules, the effective diameter of the Hydrowell^®^ module of 2.5 in was assumed. Hence, the module volume was assumed to be 0.30 m × (6.35/2)^2^ = 0.95 × 10^−3^ m^3^ [31]. The estimated *W*_max_^vol^ of the SW-FO module of Hydrowell^®^ [28] and SW PRO [29] were 0.40 kW/m^3^ and 0.75 kW/m^3^, respectively. These values were less than that of the HF module with the intrinsic *A*-value of 0.08 LMH/bar used in this study at the *C*_DS_ > 0.4 M, even though the water permeability of both SW modules was about 10 times higher than that of the HF module. This is because the HF membrane module has about eight times higher packing density (*S*_m_/*V*_mod_) than that of the SW modules. Moreover, an appropriate flow pattern, especially in the FS-side also would increase the PRO performance of the HF module, which is different from the SW module case because the FS side central partitioning wall for the SW FO module is absent in the case of HF FO module [30]. Therefore, these results clearly indicated that the CTA HF module was more efficient than the SW module, despite much lower membrane performance.

To calculate the *W*^conv^_max_^area^, the conversion coefficient was estimated using the packing density of a typical commercial SWRO module, because there is no commercial SW module for PRO application. Since the packing density of the current developing SW module for PRO is less than that of the commercial SWRO SW module due to the presence of additional glue lines along the central line of the membranes, the overestimated value in the module factor and packing density of the SW module led to an underestimation in the conversion coefficient for the HF module in the current study, and provides a fair comparison between the module performance with different configurations. For a more precise comparison of the respective module performances with different configurations in PRO, the volumetric-based power density (*W*_max_^vol^) can also be used for the designing of a full scale PRO plant as well as the membrane area-based power density (*W*_max_^area^).

## 4. Conclusions

In this study, PRO performance of a 5-inch scale CTA-based HF membrane module was evaluated under a wide range of operating conditions (0.0–6.0 MPa of applied pressure, 0.5–2.0 L/min FS inlet flow rate, 1.0–6.0 L/min DS inlet flow rate and 0.1–0.9 M draw solution (DS) concentration) by using a PRO/RO hybrid system. The subsequent RO system for DS regeneration enabled the evaluation of the steady stated module performance in a wide range of operating condition. In the case of pilot-scale module operation, since the DS dilution and the feed solution (FS) up-concentration must have occurred and are not negligible, unlike the lab-scale experiment, PRO performance strongly depended on not only the operating conditions and subsequent factors such as DS dilution ratio and permeation ratio, but also the FS-side ICP and DS-side ECP in all flow rate conditions in this study.

The applied hydraulic pressure difference at the maximum power density (Δ*P*_Wmax_) and the half value of the applied hydraulic pressure difference where the water flux became zero (1/(2Δ*P*_Jw=0_)) were almost the same as each other, and were approximately 70–85% of the half value of the theoretical osmotic pressure difference (Δ*π*/2) with different DS concentrations. This unfavorable reduction occurred even at sufficiently low *η* and *Φ*, and therefore, was mainly due to the presence of ICP.

To compare the module performance with different configurations, we proposed a converted parameter in which a difference of the packing density between the SW and HF module is fairly considered. In the case of the HF configuration, because of high packing density, the volumetric-based performance was higher than that of SW module, that is, the required number of modules would be less than that of an SW module in a full-scale PRO plant.

## Figures and Tables

**Table 1:** Specifications and fundamental properties of the test module in the study [31].

CTA-HF Module A	
Material	Cellulose triacetate
Configuration	Radial flow with crossly wounded HF config.
Number of ports	4
Module (Element) diameter	176 (136) mm
Module (Element) length	825 (683) mm
Inner diameter of hollow fiber	60 µm
Outer diameter of hollow fiber	160 µm
Number of hollow fibers	214,000
Membrane area	72 m^2^
Water permeability *A*	0.09 LMH/bar
Salt permeability *B*	0.029 LMH

**Table 2:** Comparison of the PRO module performance with different configuration.

Module	Type	Diameter		Length		Mod. vol., V_mod_	S_m_	S_m_/V_mod_	C_DS_	T	ΔP at W_max_	W_max_^area^	W^conv^_max_^area^	W_max_^vol^	Ref
		(inch)	(mm)	(inch)	(mm)	(m^3^)	(m^2^)	(1/m)	(M)	(°C)	(bar)	(W/m^2^)	(W/m^2^)	(kW/m^3^)	
Hydrowell^®^	SW	2.5 *	63.5 *	11.8	300	9.50 × 10^−4^ *	0.94	989 *	0.50	22–24	4.5	0.40	-	0.40 *	[28]
SW PRO	SW	7.9	200	39.4	1000	3.14 × 10^−2^	29	923	0.52	25	9.8	0.81	-	0.75	[29]
CTA HF (A)	HF	5.4	136	25.1	638	9.27× 10^-3^	72	7769	0.10	25–27	1.1	0.00	0.02	0.02	this study
									0.20	25–27	3.3	0.03	0.17	0.20	this study
									0.30	25–27	5.3	0.05	0.32	0.38	this study
									0.40	25–27	7.9	0.10	0.65	0.78	this study
									0.50	25–27	9.4	0.14	0.93	1.09	[31]
									0.60	25–27	11.4	0.17	1.13	1.36	this study
									0.70	25–27	14.4	0.27	1.73	2.08	this study
									0.80	25–27	16.8	0.35	2.28	2.74	this study
									0.90	25–27	18.0	0.44	2.82	3.39	this study

* Assumed value for comparison.
